# The impact of motivational interviewing to reduce alcohol use among adults in treatment for depression

**DOI:** 10.1186/1940-0640-8-S1-A65

**Published:** 2013-09-04

**Authors:** Derek D Satre, Stacy A Sterling, Amy Leibowitz, Wendy Lu, Constance Weisner

**Affiliations:** 1 University of California, San Francisco, Department of Psychiatry, San Francisco, CA, USA; 2 Kaiser Division of Research, Oakland, CA, USA

## 

This study examined the effectiveness of Motivational Interviewing (MI) to reduce hazardous drinking among patients in treatment for depression. The sample consisted of 307 participants ages 18 and over in depression treatment in Kaiser Permanente outpatient psychiatry who reported hazardous drinking, illegal drug use or misuse of prescription drugs in the prior 30 days, and who scored ≥ 5 on the Patient Health Questionnaire (PHQ-9). Participants were randomized to receive either 3 sessions of MI or printed literature about alcohol and drug use risks, as an adjunct to usual outpatient depression care. Preliminary analyses examined baseline group differences and outcomes among the 264 participants who completed 3- month follow-up to date. In the 3 month follow-up sample, both groups showed reduction in hazardous drinking and depression. Among participants reporting any hazardous drinking at the 4+/5+ level at baseline (N=154), MI-treated participants were less likely than controls to report hazardous drinking at 3 months, at a level approaching statistical significance (44.9% vs. 59.2%, p = .075). No group differences in cannabis use reduction or depression reduction were observed. This study examined preliminary data regarding the efficacy of MI (intervention) versus printed literature on alcohol use risks (control) among depression patients in a clinical setting as a supplement to usual care in psychiatry. Initial findings indicate that both groups reduced hazardous drinking at 3 months, but that MI may be somewhat more effective among participants drinking at a 4+/5+ level. The investigators are in process of completing 3-, 6- and 12-month follow up. Further analyses will examine long term outcomes, outcomes among subgroups based on drinking and psychiatric severity, predictors of hazardous drinking reduction in the sample as a whole, initiation with specialty alcohol and drug treatment among dependent participants, and cost effectiveness of the intervention.

